# Necdin modulates proliferative cell survival of human cells in response to radiation-induced genotoxic stress

**DOI:** 10.1186/1471-2407-12-234

**Published:** 2012-06-12

**Authors:** Julie Lafontaine, Guergana Tchakarska, Francis Rodier, Anne-Marie Mes-Masson

**Affiliations:** 1 Centre de recherche du Centre hospitalier de l’Université de Montréal (CRCHUM), Institut du cancer de Montréal, Y-4606, 1560, rue Sherbrooke Est, Montréal, QC, H2L 4 M1, Canada; 2 Département de Radiologie, Radio-oncologie et médecine nucléaire, Université de Montréal, Montréal, QC, H3C 3J7, Canada; 3 Département de médecine, Université de Montréal, Montreal, QC, H3C 3J7, Canada

**Keywords:** Necdin, p53, Senescence, Radioresistance, IMR90

## Abstract

**Background:**

The finite replicative lifespan of cells, termed cellular senescence, has been proposed as a protective mechanism against the proliferation of oncogenically damaged cells, that fuel cancer. This concept is further supported by the induction of premature senescence, a process which is activated when an oncogene is expressed in normal primary cells as well as following intense genotoxic stresses. Thus, deregulation of genes that control this process, like the tumor suppressor p53, may contribute to promoting cancer by allowing cells to bypass senescence. A better understanding of the genes that contribute to the establishment of senescence is therefore warranted. Necdin interacts with p53 and is also a p53 target gene, although the importance of Necdin in the p53 response is not clearly understood.

**Methods:**

In this study, we first investigated Necdin protein expression during replicative senescence and premature senescence induced by gamma irradiation and by the overexpression of oncogenic RasV12. Gain and loss of function experiments were used to evaluate the contribution of Necdin during the senescence process.

**Results:**

Necdin expression declined during replicative aging of IMR90 primary human fibroblasts or following induction of premature senescence. Decrease in Necdin expression seemed to be a consequence of the establishment of senescence since the depletion of Necdin in human cells did not induce a senescence-like growth arrest nor a flat morphology or SA-β-galactosidase activity normally associated with senescence. Similarly, overexpression of Necdin did not affect the life span of IMR90 cells. However, we demonstrate that in normal human cells, Necdin expression mimicked the effect of p53 inactivation by increasing radioresistance.

**Conclusion:**

This result suggests that Necdin potentially attenuate p53 signaling in response to genotoxic stress in human cells and supports similar results describing an inhibitory function of Necdin over p53-dependent growth arrest in mice.

## Background

The maintenance of genomic integrity relies on the ability of the p53 tumor suppressor to arrest the cell cycle thereby allowing correct repair of potentially oncogenic DNA damage after an insult [1]. This checkpoint is central to prevent the accumulation of mutations in cells, which could result in carcinogenesis. Another level of protection preventing carcinogenesis is cellular senescence, a process that also involves p53 [2,3]. Replicative senescence in human cells results from telomere shortening as a consequence of each cell division [4]. This naturally occurring process could be related to the aging of mammals as the accumulation of senescent cells may contribute to reduce tissue functionality and may affect its morphology [3]. On the other hand, stress-induced senescence also defines a permanent growth arrest that is triggered by irregular signaling in a cell caused by an activated oncogene or an unresolved genotoxic stress [5-8]. Replicative senescence and stress-induced senescence result from common mechanisms in which the tumor suppressors Rb and p53 play a central role [9]. Senescent cells remain metabolically active, with a flat morphology and are characterized by β-galactosidase activity [10]. Some effectors of p53 such as the inhibitor cyclin-dependent kinase p21, miR34 and PML are well characterized for their involvement in this permanent growth arrest [11-13]. However, new targets of p53 are continuously discovered and require extensive characterization to entirely understand their functions in the p53 pathways that regulate cell cycle, apoptosis and senescence. Moreover, p53 regulation is complex and remains incompletely understood [14].

Necdin has been recently identified as a p53 target gene [15,16]. We initially observed that Necdin is increased in polyomavirus large T-antigen expressing NIH3T3 cells, a mouse model used to unravel early events in carcinogenesis [16]. In this model, the increase in Necdin results from a p53-independent mechanism suggesting that other mechanisms are involved in Necdin transcriptional regulation. Necdin was first described as a growth suppressor with an Rb-like activity by interacting with E2F1 to repress its function [17-19]. However, we observed that NIH3T3 cells could grow efficiently even with high Necdin expression [16]. The role of Necdin in cancer remains poorly defined. A decrease in expression is observed in melanomas [20], while pancreatic cancer presents increased expression through the loss of imprinting in the Necdin gene [21]. We hypothesized that Necdin expression could be associated with better outcomes, as suggested since Necdin is associated with a better prognosis in breast cancer [22] and by our previous results revealing that Necdin expression is limited to borderline ovarian cancer, which is usually p53 wild type cancer [16].

Necdin is linked to p53 pathways suggesting that it may impact cancer development. The initial evidence by Taniura and al. [19], demonstrated a possible interaction between Necdin and p53. It was also reported that Necdin has the ability to suppress p53-induced apoptosis [19,23]. We previously observed that Necdin expression allowed cells to overcome the growth arrest induced by p53 activation [16]. In addition, Necdin can affect posttranslational modification of p53 by interacting with Sirt1 [23]. Sirt1 is a deacetylase [24] and the interaction of Sirt1 with Necdin potentiates its capacity to decrease p53 acetylation leading to the reduction of p53 activity [23]. Therefore, Necdin seems to be closely related to p53 since it is a p53 target gene and it can negatively modulate p53-dependent growth arrest and apoptosis. Its relationship to p53 activity needs to be further clarified. These observations prompted us to explore the possible impact of Necdin during senescence, another important role of p53 in preventing cancer. Here we demonstrate that while Necdin expression levels decline in both replicative and premature senescence, modulation of its expression does not affect human primary cell life span. However, high levels of Necdin contribute to an increased radioresistance in primary cells. Taken together, these observations are consistent with an inhibitory effect of Necdin on the p53 pathway and suggest a role for Necdin, under stress conditions, in preventing senescence induction.

## Methods

### Cell culture

Low passage IMR90 normal human fibroblasts [25] were a generous gift from Dr. Christian Beausejour (Centre de recherche du CHU Ste-Justine, Montreal). Primary cells were kept at 37°C under 5% CO_2_ and a low O_2_ condition (5%), and were maintained in Dulbecco’s modified eagle medium (DMEM) supplemented with 10% FBS. The number of living cells was determined using the CASY® cell counter model TT. Proliferation of IMR90 cells was assessed by population doubling according to the following formula: last PD + (Log (final cell number) - Log (initial cell plated) x 3.32).

### Vectors and infection

All constructs for lentivirus production were derived from a previously described expression system [26]. The human *NDN* gene was excised from pOBT7 containing full length human *NDN* (Open Biosystems, MHS1011-61084) and inserted in the 686–1 vector (pENTR4 no ccdB, Addgene number 17424). Recombination of this construct was performed with the destination expression vectors 685–3 or 670–1 (pLenti CMV/TO Neo DEST and pLenti CMV/TO Puro DEST: Addgene number 17292 and 17293) using the Gateway LR Clonase® enzyme mix (Invitrogen). Control vector was generated by recombination of the empty pENTR4 no ccdB with the same destination vectors. GSE22 (encoding an interfering p53 fragment) have been previously described [27]. H-RasV12 in 685–3 vector was a gift from Christopher Wiley (from J. Campisi’s lab). pLKO.1 lentiviral shRNA vectors targeting human *NDN* gene (shNdn1 (TRCN0000020085), shNdn2 (TRCN0000020086)) or GFP as a control (shGFP (RHS4459)) were purchased from Open biosystems.

Lentiviruses were produced by co-transfection of the different pLenti contructs together with ViraPower Lentiviral Packaging Mix (Invitrogen) in the 293FT packaging cell line. 72 hrs later, supernatants were collected and viruses were concentrated by ultracentrifugation. Infections were performed on 5–7.5 x 10^5^ cells overnight in the presence of polybrene. Appropriate selection was applied 48 hrs later.

IMR90 cells expressing the tetracycline repressor (TetR) were generated by infection with lentiviruses containing the 716–1 vector (pLenti-CMVtetR Blast, Addgene #17492).

### Senescence

Senescent IMR90 cells were generated by irradiating cells at 20 Gy in a Gammacell irradiator. Cell extracts were harvested at indicated times for western blot analysis. For oncogene-induced senescence, IMR90 cells were infected with oncogenic RasV12 containing lentivirus. Senescence was assessed by senescence-associated ß-galactosidase (SA-ß-gal) staining [10] using a Senescence Detection Kit (BioVision) in 6-well or 12-well culture plates according to the manufacturer’s instructions. Cells were plated 24 hrs before staining.

### Growth arrest and FACS

For serum starvation, cells were washed 24 hrs after plating and medium was replaced by DMEM containing 0.1% FBS. After 24 hrs of exposure to low or normal serum conditions, cells were collected, fixed with ethanol and stained with propidium iodide. DNA content analysis was performed using a Coulter EPICS XL-MLC Flow Cytometer.

### Western blotting

Cells were lysed with a buffer containing 50 mM Tris HCl, pH 7.4, 150 mM NaCl, 1 mM EDTA, 1% TRITON™ X-100 and protease inhibitors (Complete Protease Inhibitor Cocktail Tablets, Roche). Western blot analyses were done on nitrocellulose membranes hybridized with various antibodies: from Santa Cruz p53 (DO-1, sc-126), p21 (F5, sc-6246), H-Ras (F-235, sc-29), p16 (JC-8, sc-56330), PCNA (FL-261, sc-7907), from Millipore (Upstate) Necdin (07–565) and from Abcam β-actin (AC-15, ab6276). Secondary HRP-conjugated antibodies were all purchased from Santa Cruz.

### p53 stimulation

One day after 1 x 10^5^ cells were seeded in 6-well plates they were treated for 24 hrs with 2.5 μM Nutlin-3 (Sigma) or DMSO for the untreated control. Cells were collected and total protein extracts were performed in the lysis buffer as described above. p53 accumulation was assessed by Western blot.

### Colony formation assay

For stress-induced senescence analysis, we first transduced IMR90 cells containing the tetracycline repressor (IMRtetR) with the vector of interest (empty vector and hNdn) and IMR90 cells with GSE22 or shRNA (shNdn1, shNdn2 and shGFP). After an appropriate period of selection, cells were exposed to irradiation. For each population, 100 untreated cells were seeded to determine plating efficiency. 1.6x10^3^ cells irradiated with a dose of 2 Gy were plated in 100 mm. All conditions were performed in triplicate. Medium was replaced every third day and cells were stained after 12 days with a crystal violet solution. Percentage of colonies was determined by the following formula: number of colonies in irradiated cells/(number of colony in untreated cells x dilution factor). Since Necdin expression was inducible, we chose to maintain all populations in this experiment in medium containing doxycycline to avoid variation.

## Results

### Necdin and p53 expression are linked in human fibroblasts

**Figure 1:**
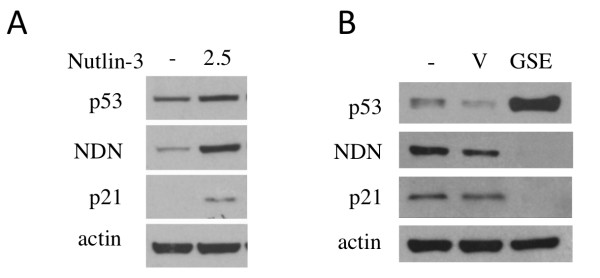
**Expression of Necdin is related to p53 activity in primary human fibroblasts.** (**A**) Stimulation of IMR90 cells for 24 hrs with Nutlin-3 (2.5 μM) to induce p53 resulted in increase in Necdin protein level (**B**) Necdin, p21 and p53 protein expression in IMR90 cells expressing GSE22 inhibitor peptide.

Physical and functional interactions between Necdin and p53 have been reported in both mouse and human cancer cell lines [15,16,19]. It was of interest to determine if this link can also be found in normal human primary cell line. In order to induce p53, human primary IMR90 fibroblasts were treated with Nutlin-3, a mdm2 antagonist that causes an accumulation of p53 in cells [28]. Nutlin-3 treatment induced an accumulation of p53 at the protein level (Figure 1A). Concomitant with p53 induction, the increase in Necdin occurred in conjunction with increased p21, a known p53-target gene (Figure 1A). Inversely, when a dominant negative peptide suppressor of p53 activity, GSE22 [29,30], was transduced in IMR90 using a lentivirus construct, we observed a decrease in both Necdin and p21 protein levels (Figure 1B). The presence of GSE22 in cells is well known to induce the accumulation of a non-functional p53 as a result of interaction of the peptide with p53 (Figure 1B). These results are consistent with earlier results in cancer cell lines, and demonstrate a link between Necdin and p53 epression in human primary cells.

### Necdin level decreases with replicative senescence

**Figure 2:**
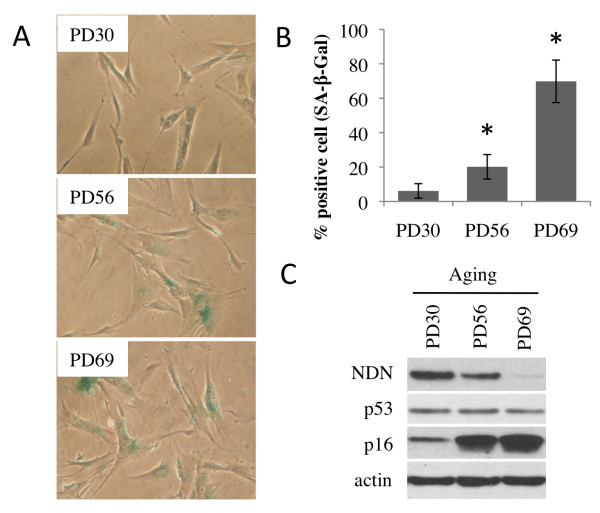
**Necdin levels decrease during replicative aging in primary cells.** (**A**) Representative micrographs of senescence-associated β-galactosidase staining of IMR90 cells at early passage (PD30), late passage (PD56) and senescence (PD69). (**B**) Quantification of senescent cells represented by the average of SA-β-gal positive cells. Error bars represent standard deviation of counts performed on six different fields. (**C**) Western blot analysis of protein content from IMR90 cells at corresponding population doubling. All extracts were collected the same day from the three different populations.

To investigate the connection between Necdin and cellular senescence, we first characterized Necdin expression during the normal process of replicative aging of cells *in vitro*. Human primary fibroblasts (IMR90) were maintained in culture for various periods of time thereby generating populations at three different population doubling (PD). Young (PD30), late passage (PD56) and senescent (PD69) fibroblasts were characterized for senescence-associated β-galatosidase activity (SA-β-Gal) (Figure 2A and B), a biomarker of senescence [10]. Young and presenescent fibroblasts showed low staining (6% and 20% respectively) while the older cell culture had up to 70% SA-β-Gal of positive cells (Figure 2B). When these cells were compared at the protein level, a decrease in Necdin expression that correlated with cellular aging was clearly observed. Necdin was present in young cells, moderately in later passages and barely detectable in senescent cells (Figure 2C). This variation was accompanied by a characteristic upregulation of the cell cycle inhibitor p16^INK4A^, which was noticeable in late passage cells and sustained in senescent cells. There was no difference in p53 levels between the various populations (Figure 2C). These results revealed that Necdin expression declines when cells reach replicative senescence.

### Premature senescence is accompanied by a decrease in necdin level

**Figure 3:**
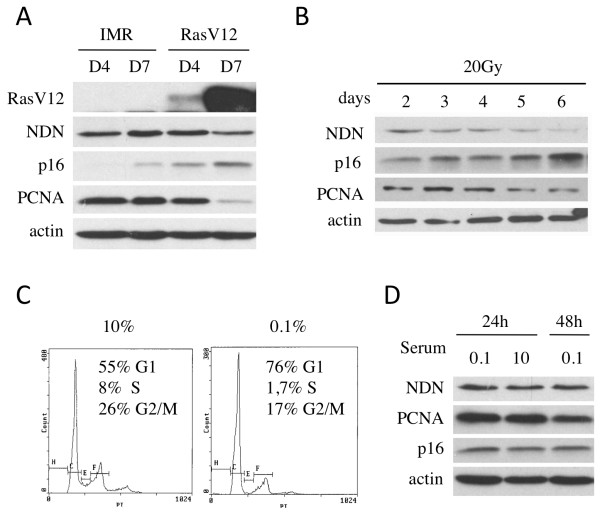
**Premature senescence is characterized by Necdin downregulation.** (**A**) Oncogene-induced senescence induced by RasV12 transduction of IMR90 cells caused a decrease in Necdin levels. Protein extracts were collected at the proliferation stage (day 4) and senescence (7 days after infection) (**B**) Stress-induced senescence generated by irradiation of IMR90 cells with 20 Gy also resulted in decreased Necdin level. Western blot analysis revealed protein modulation over time. (**C**-**D**) Necdin variation is not observed with transient arrest induced by serum starvation. (**C**) Flow cytometry analysis of IMR90 cells showed growth arrest after 24 hrs of serum starvation (0.1% FBS) compared to normal culture conditions (10% FBS). (**D**) Necdin protein levels of cells in normal or low serum conditions did not change.

To further characterize Necdin expression during senescence, we also followed its expression in situations where senescence was induced in response to a cellular insult. Premature senescence is triggered when an oncogene is overexpressed or present in an activated form in a cell, and is thought to represent a fail-safe mechanism to prevent carcinogenesis [5,31]. This senescence is known as oncogene-induced senescence (OIS). We used a human activated mutant form of Ras (Ha-RAS^v12^) [32], which induces OIS soon after its expression in normal human fibroblasts when expressed alone [5]. After transduction of oncogenic RasV12, cells usually grew for a short period of time before entering into a permanent growth arrest [33,34]. Different time points were selected after infection to evaluate protein content during the proliferation stage (day 4) and in senescent cells (day 7). The transduction of RasV12 resulted in high levels of Ras at day 7 compared to control cells where endogenous Ras was not detected (Figure 3A). However, four days after infection, the level of Ras was more moderate (Figure 3A). Transduction of RasV12 did not induce a variation of Necdin during the proliferation stage (day 4), but the Necdin level diminished when cells reached senescence one week after infection (Figure 3A). As expected, p16^INK4A^ levels gradually increased from day 4 in RasV12 expressing cells compared to control. PCNA was included as a readout of the proliferative capacity of the different populations and showed a marked decrease consistent with growth arrest at day 7 in the RasV12-transduced population.

Another method to induce premature senescence in human normal cells is exposure to stresses causing persistent DNA damages [7]. In order to induce stress-induced premature senescence (SIPS), cells were exposed to gamma irradiation. Cells that were irradiated at 20 Gy progressively entered a senescence state, which was marked by an increase in p16^INK4A^ expression over time (Figure 3B). This increase in p16^INK4A^ was accompanied by a decrease in Necdin protein levels (Figure 3B). Finally, we evaluated whether the decrease in Necdin levels observed with senescence was also seen in transient growth arrest. Cells were subjected to serum starvation for either 24 or 48 hours, which induced a cell cycle arrest in G1 as confirmed by DNA content (Figure 3C). Western blot analysis revealed a constant level of Necdin protein during this transient growth arrest (Figure 3D).

Thus, during both oncogene-induced (RasV12) and stress-induced (irradiation) senescence, Necdin levels decreased with the establishment of senescence. The decrease in Necdin expression is specific to permanent growth arrest since serum depletion did not generate the same effect.

### Modulation of necdin does not affect replicative aging

**Figure 4:**
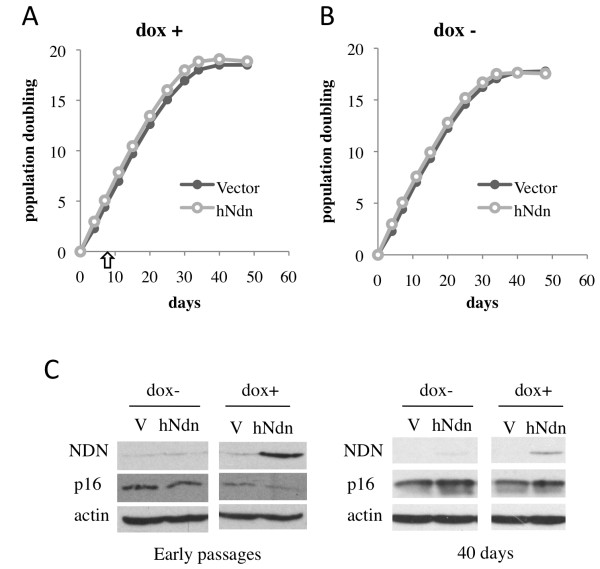
**The effect of Necdin overexpression on cellular life span.** Population doubling of an IMR90tetR population expressing inducible Necdin or empty vector (**A**) with or (**B**) without doxycycline induction (arrow in A indicates the time where induction began). Population doubling monitoring begins after the selection period where cells achieve PD40 (corresponding to PD0 on the graph). (**C**) Protein extracts at two different time points showed efficient Necdin induction with doxocyclin. Representative results of three independent experiments.

These results raised the possibility that Necdin may have an inhibitory effect on senescence or that the decrease in Necdin may be a consequence of senescence. Thus, it was of interest to determine if sustained Necdin expression could allow cells to extend their proliferative lifespan. Lentiviruses were used to deliver human Necdin cDNA in IMR90 cells. Because we previously observed a short-term growth inhibition with Necdin expression in the mouse NIH3T3 immortal cell line [16], we used an inducible system to express Necdin in IMR90 expressing the tetracycline repressor (IMRtetR). This inducible system allowed us to assess the impact of Necdin expression upon induction after an extended period of selection due to the low titer of viruses produced from Necdin cDNA. Population doublings were followed until cells naturally reached replicative senescence. Upon induction by doxycycline, a more stable analogue of tetracycline, we did not observe any effect of Necdin overexpression on the growth rate of IMR90 cells (Figure 4A). Moreover, IMR90 cells expressing Necdin did not present an extended life span compared to vector expressing control as both populations arrested after the same number of population doubling (Figure 4A). Neither proliferation nor life span was affected by the presence of doxycycline (Figure 4A and B). Induction of Necdin expression by doxycycline was monitored at different time points. As presented in Figure 4C, ectopic expression of Necdin was maintained even when cells are arrested although with a lower efficiency than seen in early passages.

**Figure 5:**
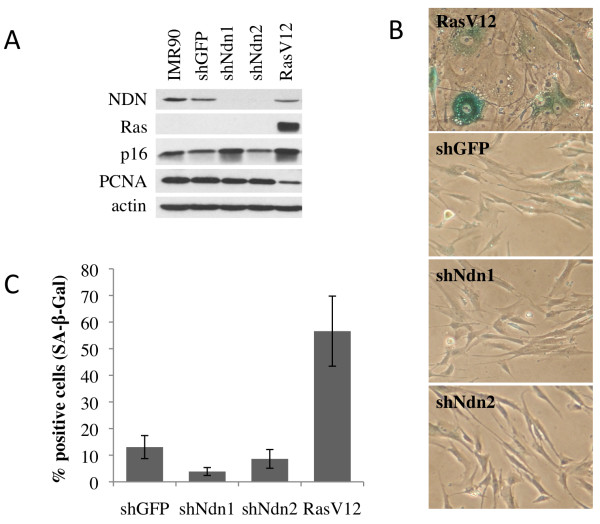
**The effect of Necdin depletion on premature senescence.** (**A**) Protein expression level in IMR90 cells showed efficient depletion in Necdin expression by shRNA and overexpression of RasV12, which served as a positive control of senescence induction. (**B**) Senescence-associated β-galactosidase staining 14 days after lentiviral infection demonstrated senescence only in RasV12-expressing cells. (**C**) Quantification of senescent cells represented by the average of SA-β-gal positive cells. Mean of six different fields ± standard deviation.

Inversely, we also analyzed the effect of decreasing Necdin expression in IMR90 cells. Protein level analysis confirmed a significant reduction in Necdin by using two distinct shRNA delivered by lentiviruses (Figure 5A). A vector containing shRNA against GFP served as negative control and the oncogene RasV12 served as positive control for premature senescence (Figure 5A). Elevation of p16 was visible in RasV12 expressing cells at 7 days after infection as well as a modest increase in one of the two populations where shNdn were expressed (Figure 5A). However, expression of RasV12 did induce premature senescence phenotype as showed by flat morphology and determined by SA-β-galactosidase staining but neither of the shRNA population presented these characteristics (Figure 5B). Quantification of SA-β-gal staining revealed that, as expected, a significant portion of cells was positive with RasV12 expression (more than 50%) compared to control shGFP (13%) and cells expressing shNDN showed a similar or decrease in SA-β-galactosidase staining (3% and 8%) compared to negative control shGFP (Figure 5C). Take together, these results suggested that, under normal conditions, positive or negative variations in Necdin expression did not affect the cellular proliferation or the life span of normal human IMR90 cells.

### Necdin overexpression provides radio-resistance in human normal cells

**Figure 6:**
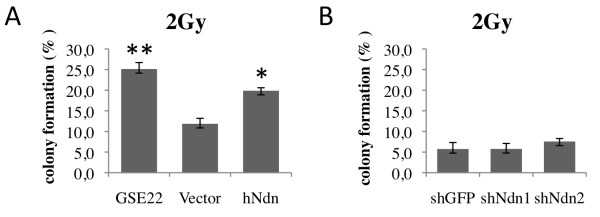
**Necdin overexpression confers radioresistance.** Colony formation assays were performed on human fibroblasts exposed to 2 Gy of ionizing irradiation. Percentage of recovery was evaluated 12 days after irradiation in (**A**) IMR90tetR cells overexpressing human Necdin, empty vector and GSE22 or (**B**) IMR90 cells where Necdin expression was targeted by two different shRNAs compared to control shGFP. Mean ± standard deviation. (*P = 0.02, *t*-test).

Although Necdin did not appear to affect senescence in normal conditions despite its involvement in Rb-E2F1 and p53 pathways, it was of interest to determine if Necdin expression could modify the cellular response to stress-induced senescence. To characterize the effect of Necdin modulation under stress conditions, cells where Necdin was overexpressed or targeted by shRNA were irradiated with a dose of 2 Gy to induce DNA damage. Under these conditions, DNA double-strand breaks (DSBs) are induced in all nuclei [35] and p53 is induced (Additional file 1). A single-cell colony formation assay after irradiation revealed the proliferative survival potential of each population. As a control, the inhibitor of p53 (GSE22) was expressed as p53 inactivation in normal cells can result in radioresistance [8]. Colony quantification showed an increase in proliferation efficiency when p53 was inactivated with proliferative survival of 25% compared to only 12% in control cells (Figure 6A). Similarly, expression of Necdin resulted in 20% of proliferating colonies suggesting a similar effect to p53 inactivation (Figure 6A). No significant variation was observed when Necdin expression was depleted by shRNA (shGFP 6%, shNdn 6% and 8%). These data suggest that Necdin overexpression in human cells increases their resistance to irradiation thus supporting the possible inhibitory activity of Necdin over a p53 function.

## Discussion

### Low necdin expression as a marker of senescence

Replicative senescence marks the end of the proliferative state in cells when telomeres reach a critical length [4]. This mechanism is relevant in limiting aberrant proliferation and may contribute to cancer prevention [36]. By comparing IMR90 cells at high or low population doublings, we demonstrate a reduction of the Necdin protein levels over time, while p16 increases, which is the mark of an irreversible arrest [29,37,38]. Lee and al. [39] identified Necdin among the gene expression profiles in skeletal muscle of aging mice, where Necdin levels decrease with age. In contrast, Necdin was upregulated upon caloric restriction, a process that retards aging in mice [39]. Our result confirmed that the decreased Necdin levels seen in aged mouse skeletal muscle can be reproduced with normal human fibroblasts in culture. The decrease in Necdin expression was also consistently observed in telomere-independent premature senescence induced by irradiation. It was also observed, but to a much lower extent, in premature senescence resulting from the expression of the activated oncogenic Ras (RasV12). Although the observed reduction in Necdin protein was subtle, another group has independently reported that Necdin mRNA was part of the list of downregulated genes following Ras-induced senescence [34], supporting our general finding that Necdin is downregulated during cellular senescence.

In contrast to senescence, cellular quiescence is a reversible cell cycle arrest since cells may re-enter the cell cycle when the restriction is removed [29,37]. Interestingly, in a transient G1 arrest, Necdin remained expressed, suggesting that the decrease in Necdin is limited to permanent growth arrest and may be a marker of senescent cells. Necdin levels in other primary cells need to be analyzed since the molecular signature at senescence largely depends on the cell type [40].

Like Necdin, many genes involved in cell cycle progression show altered expression in senescent cells as both proliferation-promoting proteins and their negative regulators decrease when human fibroblasts reach senescence, as exemplified by Rb and E2F1 [34,41]. Necdin interacts with E2F1, E2F4 and also affects expression of the Rb family of genes [42]. One interpretation of these findings is that Necdin may affect cellular senescence. To test this hypothesis, ectopic expression of Necdin was used. We found that overexpression of Necdin did not extend the replicative life span of primary human fibroblasts. Moreover, the sustained expression of Necdin over time in this experiment suggested that the complete elimination of Necdin expression is not essential for senescence to occur.

There are various examples in the literature where proteins, possessing properties similar to Necdin, can induce premature senescence when depleted in IMR90 cells. The knock down of BS69 and p400, also known to interact with viral proteins, induced a premature senescence characterized by p21 and p53 upregulation [43,44]. BS69 and p400, like Necdin, can form a complex with p53 at the p21 promoter to repress transcription [19,44], although it is not known if Necdin is part of the same complex. Moreover Sirt1, a partner of Necdin [23], can increase the risk of cancer due to its capacity to downregulate p53 activity by deacetylating this tumor suppressor [24,45,46]. Accordingly, Sirt1 inhibition also induces a senescence-like phenotype in IMR90 cells [47]. From these data, we expected that knock down of endogenous Necdin by shRNA might also induce premature senescence resulting from p53 activation and p21 de-repression. However, shNDN-directed loss of Necdin expression did not induce premature senescence in IMR90 cells. Perhaps, under non-stress conditions, a complementary protein may contribute to maintaining Necdin function in its absence. For example, other members of the MAGE family like NDNL2, also known as MAGE-G1, [48], are expressed in a wide variety of tissues including fibroblasts [49] and shares many functions with Necdin. Alternatively, loss of Necdin might not be sufficient to activate p53 in the absence of others p53 stabilization signals.

Necdin is a maternally imprinted gene and its promoter contains many CpG sites for methylation regulation [50]. Consistent with this, the inhibition of DNA methyltransferase (DNMT) by 5-aza-2′-deoxycytidine (5AZA-dC) has been shown to induce Necdin expression in some cancerous cell lines [51]. During senescence establishment, epigenetic changes occur inducing important chromatin structure modifications; some at specific sites while other reflect a more global change. Global DNA methylation status decreases with aging by comparison to young counterparts and immortalized cells [52,53]. A specialized redistribution in chromatin heterochromatin, called senescence-associated heterochromatic foci (SAHF), is also associated with cellular senescence [33]. The mechanism is only partially understood; the genomic loci affected by this structure often contain proliferation-promoting genes such as E2F1 target genes [33]. Moreover, SAHF are not observed in quiescent cells. It is possible that the decrease in Necdin levels during aging could be the result of hypermethylation or others senescence associated modifications at specific sites.

### Necdin expression confers resistance to ionizing irradiation

Necdin expression can exert an effect on normal cells as an increase in Necdin level confers resistance to ionizing radiation. At the employed dose, DNA double-strand breaks were induced in all cells and the physiological consequence is the induction of a DNA damage response activating p53. Thus, these damages caused cell cycle arrest and need to be repaired before cells can resume proliferation. When damages cannot be properly repaired, apoptosis or senescence are two possible cellular outcomes. Consequently, we observed that cells unable to form colonies showed enlarged and flat morphology of senescent cells in all populations tested (data not shown). This is expected for normal fibroblasts that are relatively resistant to apoptosis upon irradiation [54]. Apoptosis was probably also induced to a low level but could not be monitored due to the low cell densities used in these experiments.

An appropriate response to genotoxic stress is based on the capacity to sense the damage, to activate the cell cycle checkpoint and to repair the damage [55]. Two principal conditions may explain an increase in resistance to irradiation in a normal cell. First, an enhanced ability to repair the DNA damage could promote survival. Second, a failure in activating cell cycle checkpoints will contribute to the maintenance of cells in a proliferative state despite the presence of a genotoxic stress. This could result from inefficient sensing upstream of the p53 pathways or by a reduction in p53 downstream signaling. This is what was reproduced with the positive control expressing a peptide inhibitor of p53 (GSE22) resulting in a marked increase in colony formation consistent with the notion that a decrease in p53 function increases resistance to irradiation. We observed and others have shown previously that Necdin can interfere with p53-responses [16,19,23]. These data suggest that increased radioresistance associated with Necdin may be related to its ability to directly influence the p53-response. Necdin may also confer radioresistance by inhibiting radiation-induced apoptosis since Necdin can negatively modulate caspase activation upon genotoxic stress [56,57], but this is unlikely in fibroblast in response to irradiation [54]. Further evidence supporting Necdin’s ability to contribute to radioresistance is a microarray analysis of the gene expression profiles produced by radiosensitive and radioresistant esophageal carcinoma cell lines. In this study, Necdin expression was higher in radioresistant cells [58] which is consistent with the observation of the present study.

## Conclusion

The results of the present study suggest that Necdin function in regulating p53 responses is revealed only under stress conditions. In the absence of exogenous genotoxic stress, Necdin has no effect on normal cellular life span in human cells.

## Competing interests

The author(s) declare that they have no competing interests.

## Authors’ contributions

The conception and design of experiments, as well as analysis and interpretation of data, was done by all authors. JL and GT performed all the experiments. The manuscript was written by JL and reviewed by FR and A-MM-M. All authors read and approved the final manuscript.

## Pre-publication history

The pre-publication history for this paper can be accessed here:

http://www.biomedcentral.com/1471-2407/12/234/prepub

## Supplementary Material

Additional file 1 **p53 induction by 2 Gy gamma-irradiation.** p53 protein levels were monitored in IMR90 at days 0 to 2, after the cells were irradiated at 2 Gy. Actin was used as loading control. Click here for file
