# Supplementary figures and images for: Necdin modulates proliferative cell survival of human cells in response to radiation-induced genotoxic stress
Source: BMC Cancer. 2012 Jun 12;12:234. doi: 10.1186/1471-2407-12-234 (PMC3495902; doi:10.1186/1471-2407-12-234)

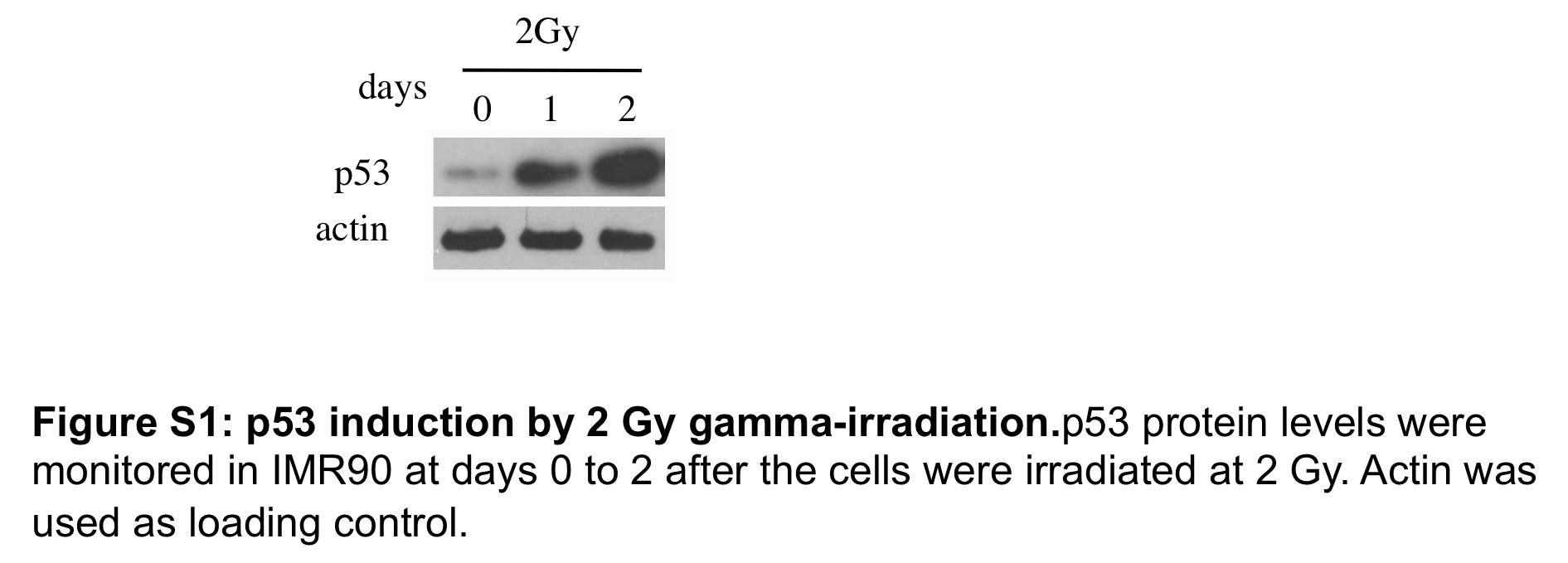

Supplement: Additional file 1 — p53 induction by 2 Gy gamma-irradiation. p53 protein levels were monitored in IMR90 at days 0 to 2, after the cells were irradiated at 2 Gy. Actin was used as loading control. [file 1471-2407-12-234-S1.tiff]
